# Automatic image segmentation based on synthetic tissue model for delineating organs at risk in spinal metastasis treatment planning

**DOI:** 10.1007/s00066-019-01463-4

**Published:** 2019-04-29

**Authors:** Olaf Wittenstein, Patrick Hiepe, Lars Henrik Sowa, Elias Karsten, Iris Fandrich, Juergen Dunst

**Affiliations:** 1 Department of Radiation Oncology, Universitätsklinikum Schleswig-Holstein Campus Kiel, Arnold-Heller-Straße 3, Haus 50, 24105 Kiel, Germany; 2 R&D Anatomical Mapping, Brainlab AG, Olof-Palme-Straße 9, 81829 Munich, Germany

**Keywords:** Radiotherapy, Extra-cranial, Automization, Elements, Atlas, Strahlentherapie, Extra-kraniell, Automatisierung, Elements, Atlas

## Abstract

**Purpose:**

One of the main goals in software solutions for treatment planning is to automatize delineation of organs at risk (OARs). In this pilot feasibility study a clinical validation was made of computed tomography (CT)-based extracranial auto-segmentation (AS) using the Brainlab Anatomical Mapping tool (AM).

**Methods:**

The delineation of nine extracranial OARs (lungs, kidneys, trachea, heart, liver, spinal cord, esophagus) from clinical datasets of 24 treated patients was retrospectively evaluated. Manual delineation of OARs was conducted in clinical routine and compared with AS datasets using AM. The Dice similarity coefficient (DSC) and maximum Hausdorff distance (HD) were used as statistical and geometrical measurements, respectively. Additionally, all AS structures were validated using a subjective qualitative scoring system.

**Results:**

All patient datasets investigated were successfully processed with the evaluated AS software. For the left lung (0.97 ± 0.03), right lung (0.97 ± 0.05), left kidney (0.91 ± 0.07), and trachea (0.93 ± 0.04), the DSC was high with low variability. The DSC scores of other organs (right kidney, heart, liver, spinal cord), except the esophagus, ranged between 0.7 and 0.9. The calculated HD values yielded comparable results. Qualitative assessment showed a general acceptance in more than 85% of AS OARs—except for the esophagus.

**Conclusions:**

The Brainlab AM software is ready for clinical use in most of the OARs evaluated in the thoracic and abdominal region. The software generates highly conformal structure sets compared to manual contouring. The current study design needs revision for further research.

## Zusammenfassung

**Zielsetzung:**

Bei der Entwicklung neuer Softwarelösungen zur Bestrahlungsplanung wird eine Automatisierung der Konturierung von Risikoorganen (OARs) angestrebt. In dieser Arbeit wurde die computertomographie(CT)-basierte automatische Segmentierung (AS) extrakranieller Strukturen des Anatomical-Mapping(AM)-Tools der Firma Brainlab validiert.

**Methode:**

Anhand der Datensätze von 24 behandelten Patienten wurde die Konturierung von 9 extrakraniellen OARs retrospektiv überprüft. Die in der klinischen Routine manuell erstellten Konturen wurden mit durch AM automatisch erzeugten Datensätzen verglichen. Als Messmethoden kamen der Dice Similarity Coefficient (DSC) und die Hausdorff Distance (HD) zum Einsatz. Zusätzlich wurden alle automatisch erzeugten Strukturen mit Hilfe eines subjektiven qualitativen Punktesystem bewertet.

**Ergebnisse:**

Die automatische Konturierung der OARs aller untersuchten Datensätze durch die AS-Software konnte durchgeführt werden. Für die Organe linke Lunge, rechte Lunge, linke Niere und Trachea zeigten sich hohe DSC-Werte mit geringer Variabilität. Bei den übrigen Organen (mit Ausnahme des Ösophagus) bewegten sie die DSC-Werte zwischen 0,7 und 0,9. Die Berechnung der HD ergab ähnlich interpretierbare Ergebnisse. In der subjektiven Bewertung wurden >85 % der automatisch erzeugten OARs akzeptiert – außer die des Ösophagus.

**Schlussfolgerung:**

Das AM-Tool der Firma Brainlab kann für die meisten untersuchten OARs der thorakalen und abdominellen Region in der klinischen Routine eingesetzt werden. Im Vergleich zur manuellen Konturierung werden hochkonformale Strukturen erzeugt.

## Introduction

Today, virtual three-dimensional planning on computed tomography scans (CT) is state of the art in radiotherapy (RT). Common techniques like intensity-modulated radiotherapy (IMRT) and volumetric-modulated arc therpay (VMAT) are increasingly used worldwide. These techniques require highly specialized treatment planning with contouring of organs at risk (OARs; [1]). The latter represents a major part of the planning workload, since commonly used slice-by-slice manual or interpolation-based semi-automatic contouring approaches are time-consuming and repetitive tasks [2, 3]. Furthermore, these approaches are associated with an interobserver variability in contouring of the OARs that is independent of the dosimetrist’s experience [4, 5]. Hence, precise, fast, and reproducible contouring methods may facilitate treatment planning. Moreover, the introduction of techniques, e.g., adaptive RT and gating, may further increase the need for repeatedly applied planning updates for the same patient [2]. The use of automatic segmentation (AS) software could compensate for the additional expenditure of time.

Different concepts of AS such as model fitting and rule-based or image registration-based approaches were proposed recently for automated OAR delineation. Their functionality was well described by Haas et al. [6]. So far, image registration-based methods that are based on a priori anatomical atlas information represent the most promising approach for clinical applications [7]. In principle, the acquired patient dataset is registered to a pre-designed atlas dataset comprising prior knowledge of the human anatomy. In a second step, the OARs are transferred from the atlas space to the patient dataset and subsequently post-processed.

A variety of commercially available software products, e.g., MIM Maestro (MIM Software Inc., Cleveland, OH, USA), Velocity (Varian Medical System, Palo Alto, CA, USA), ABAS (Elekta AB, Stockholm, Sweden), iPlan RT Image (Brainlab AG, Munich, Germany), and SPICE (Philips Medical Systems DMC GmbH, Hamburg, Germany), have been evaluated [3, 8, 9]. These software solutions offer a potential for reproducible accuracy and time-sparing for different use cases (head and neck, prostate, breast, lung). However, most of these software solutions provide different atlases for the different extracranial regions or are specialized on a single organ [10].

In this pilot feasibility study, a novel commercially available AS software (Anatomical Mapping 1.0; Brainlab AG, Munich, Germany) was evaluated, which is integrated in a specific workflow for treatment planning of spinal metastasis (including semi-automatic delineation of Clinical Target Volumes [CTV] of vertebrae). The Anatomical Mapping software is based on a versatile atlas-based Synthetic Tissue Model designed for OAR definition in thoracic, abdominal, and pelvic body regions at the same time. This method was previously introduced by Blumhofer et al. [11]. It uses the complete anatomical tissue environment in the complex extracranial region (modeling of arm positions, muscle, and subcutaneous fat proportions) and may therefore facilitate a reliable and fully automated OAR delineation. More thorough information can be found in the Methods section.

It was hypothesized that the evaluated method (a) produces highly conformal structure sets in comparison with clinically approved reference contours (RC) of the evaluated OARs (left lung, right lung, heart, liver, left kidney, right kidney, spinal cord, trachea, esophagus) and (b) is applicable for clinical usage (qualitative validation). Patients who had undergone RT for spinal metastasis were considered for this study since they typically show a large number of OARs relevant for contouring in order to prepare the RT plan.

## Methods and materials

### Patient dataset

Retrospective clinical datasets of 24 patients who had undergone RT for spinal metastasis in a single institution from November 2016 to June 2017 were consecutively investigated in this study—29 patients were screened, 24 included (Table 1). Further considerations regarding the case number were not made. The acquisition of large CT scans with most of the thoracic, abdominal, and pelvic OARs with spinal metastasis was used as screening and inclusion criterion. The study was approved by ethics committee in Kiel (D535/18). The RT treatment plans comprised conventional 3D-CRT (three-dimensional conformal radiotherapy) of one to five vertebrae as CTV including eight cases of postoperative RT and eight cases of stereotactic body radiotherapy (SBRT) for treatment of only one vertebra or even smaller CTVs (Table 1). The noncontrast CT images were acquired by using a multidetector-row spiral CT scanner (Somatom Sensation Open, Siemens Medical Solutions, Erlangen, Germany) with the patients lying in supine position. Each patient scan showed 2‑mm-thick axial slices with a mean axial coverage of 36 cm.

**Table 1:** Patient data

Parameter	Sample size
*Total Number of patients*	29
Import/export problems	4
No relevant OARs in field-of-view	1
*Included number of patients*	24
Primary radiotherapy (no surgery)	16
Postoperative radiotherapy	8
*Type of radiotherapy technique*	
3D-CRT	16
SBRT	8

Screening included all patients who had consecutively undergone RT for spinal metastasis from November 2016 to June 2017. Import/export problems occurred randomly. In one pelvic case, no relevant OARs for this investigation were found in the field of view and it was omitted. It was also documented whether contouring of OARs was done for precise SBRT (potentially better accuracy of the manual contours) or for 3D-CRT *OAR* organ-at-risk, *3D-CRT* three-dimensional conformal Radiotherapy *SBRT* stereotactc body Radiotherapy

### Manual and semi-automatic contouring

In all patients, contouring was performed during clinical routine (blinded study) with a standard contouring platform (Eclipse, Varian Medical Systems, Palo Alto, CA, USA) by two physicians-in-training and one radiation technologist. The decision to use semi-automatic tools such as the adaptive or non-adaptive two-dimensional Brush, interpolation, and the segmentation wizard was left to each contouring professional. The following regularly used OARs (based on our institutional guidelines) were selected for the investigation: left lung, right lung, heart, liver, left kidney, right kidney, spinal cord, trachea, esophagus. The number of available and evaluated RCs for different OARs is given in Table 2.

**Table 2:** Results of quantitative assessment

Organ at risk	DSC [a.u.]	HD (mm)	Sample size
Left lung	0.97 ± 0.03	20.8 ± 12.5	15
Right lung	0.97 ± 0.05	21.2 ± 10.5	15
Heart	0.78 ± 0.16	31.2 ± 10.2	7
Liver	0.80 ± 0.17	37.7 ± 13.8	5
Left kidney	0.91 ± 0.07	17.5 ± 12.9	12
Right kidney	0.81 ± 0.28	22.9 ± 12.9	12
Spinal cord	0.71 ± 0.12	21.4 ± 19.7	18
Trachea	0.93 ± 0.04	7.6 ± 6.9	5
Esophagus	0.49 ± 0.13	30.6 ± 11.4	10

DSC scores were first published in [12]

The lower DSC and higher HD for the right kidney in comparison with the left kidney are a consequence of two outliers; one of these is further described in Fig. 2

*DSC* Dice similarity coefficient, *HD* Hausdorff distance

### Software-based automatic contouring

**Fig. 1:**
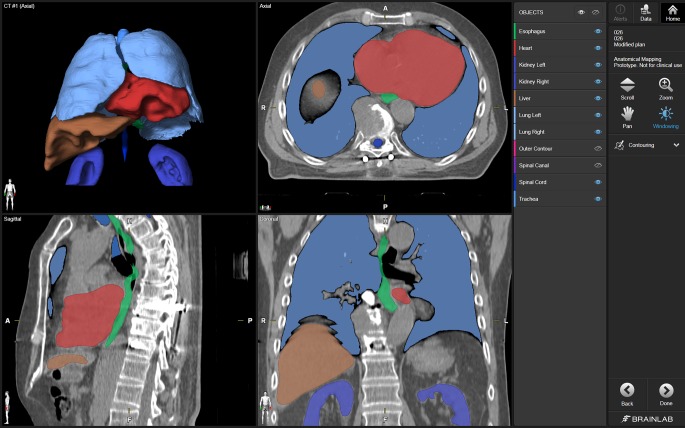
Screenshot of the Object Manipulation user interface after automatic segmentation (AS) of typical organs at risk (OARs). The computed tomography image is depicted in ACS view (axial, coronal, sagittal) with an additional 3D model (*upper left*). Visibility of OARs and the toolbar are situated on the *right side*. For illustrative purposes the visualization of AS objects was modified to show surfaces/volumes instead of contours. The visibility of two structures (Outer Contour and Spinal Canal) was switched off (*eye symbols on toolbar*)

Automatic segmentation of clinical CT images was performed by using Elements Anatomical Mapping (Release 1.0) as part of the Brainlab Elements software (Brainlab AG, Munich, Germany). Its functionality is well described by Daisne and Blumhofer [11]. Via Elements Anatomical Mapping for segmentation, the Synthetic Tissue Model (atlas) is mapped by means of nonlinear, elastic image fusions onto the patient dataset and structures outlined in the Synthetic Tissue Model are transferred to the patient dataset. During this process, the algorithm also detects anatomic variabilities (e.g., gender and patient positioning). Afterwards, image post-processing routines are applied to ultimately determine the segmentation results and the user can select the generation of the desired structure sets ([11]; Fig. 1). So far, extracranial image segmentation solely works on CT image data with or without contrast agent. None of the evaluated CT images and RCs were used prior to the study such as to train/optimize the algorithm. The evaluated software provides AS of more objects in different parts of the body. However, the present study was focused on OARs in patients with spinal metastasis only and additional OARs of the pelvic region (e.g., prostate, bladder, rectum, and hip joints) as well as segmentation of individual vertebrae, clavicle, sternum, aorta, and vena cava were due to low case numbers not considered for quantitative and qualitative evaluation.

### Quantitative and qualitative assessment

The comparison between manual (RC) and automatic segmentation (AS) was based on common and regularly calculated statistical or geometrical parameters [7–11, 13–17]: the Dice similarity coefficient (DSC) and Hausdorff distance (HD). The DSC ranges between 0 and 1 and scores greater than 0.7 can be interpreted as if the generated contours show a high grade of overlap with the RCs used. The HD in this evaluation ranges from 0 to 50 mm (owing to a shortening of calculation time the maximum HD was restricted to 50 mm). Lower distances mean better results. Both evaluations were performed using Matlab (MathWorks, Natick, MA, USA) scripts provided by Brainlab.

In order to better interpret the results obtained from the quantitative evaluation and to affirm or support the findings from a clinical perspective, an initial qualitative review was performed. This qualitative review relies on the subjective scoring system of Zhu et al. [9]. The scoring system rates every OAR per case with 1 = “useful without correction,” 2 = “useful with minor correction,” and 3 = “not useful.” The definition of minor correction was defined as editing in the Anatomical Mapping, AS being preferred over total manual contouring.

## Results

**Fig. 2:**
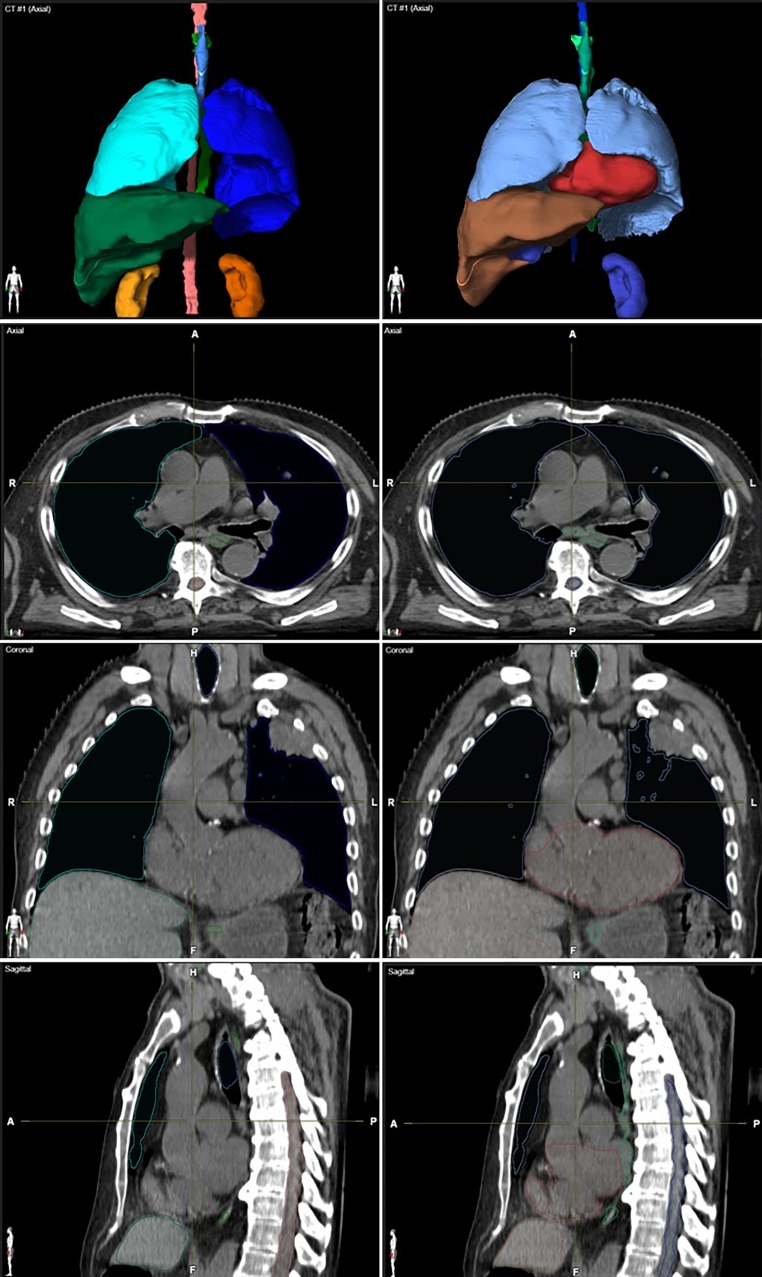
Depiction of manually and automatically generated contours of one representative dataset. Manual contours of esophagus, kidneys, lungs, liver, spinal cord, and trachea on the *left side* and automatic segmented structures on the *right side* in axial, coronal, sagittal and three-dimensional model view. The heart is segmented additionally, the missing right kidney was a segmentation failure with DSC = 0, HD = 50 mm, and qualitative score = 3. *DSC* Dice similarity coefficient, *HD* Hausdorff Distance

All patient datasets investigated were successfully processed by the evaluated AS software. Two representative patient datasets and their AS contours are shown in Figs. 1 and 2. As an example, the OARs in Fig. 1, esophagus, heart, liver, and spinal cord, were scored 2, kidneys and lungs received a qualitative score of 1. The DSCs were 0.98 for both lungs, 0.86 for the heart, and 0.56 for the spinal cord.

### Quantitative assessment

**Fig. 3:**
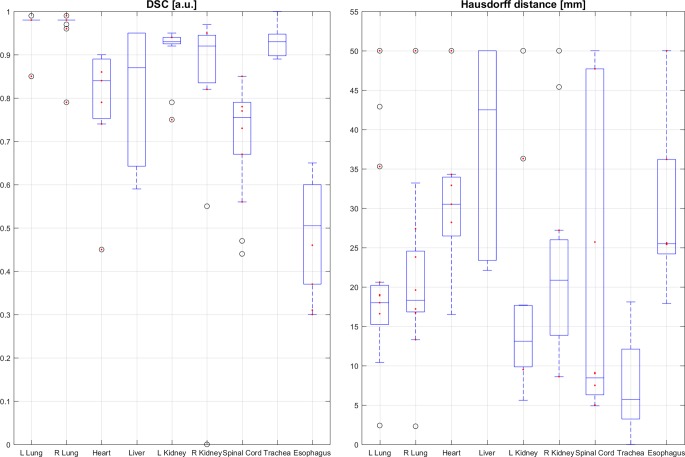
Box plots for all organs at risk evaluated. *Left side*: DSC of auto-segmented structures and reference contours, postoperative cases are marked *red*; *right side*: similar, but with maximum Hausdorff distance (AS vs. RC). *L* left, *R *right, *DSC* Dice Similarity Coefficient

Mean and SD values of the DSC and HD determined as well as the number of evaluated OARs are given in Table 2. Corresponding box plots of both similarity measures are shown in Fig. 3. The DSC box plots show the highest values with low variability for the left Lung (0.972 ± 0.034), right lung (0.967 ± 0.050), left kidney (0.908 ± 0.066), and trachea (0.930 ± 0.043). Other organs, except the esophagus, show DSC scores ranging between 0.7 and 0.9 with higher interquartile ranges (distances between the box tops and bottoms related to the 25th and 75th percentiles of the samples, respectively). The esophagus yielded the lowest mean and median DSC values of 0.486 ± 0.133 and approx. 0.5, respectively.

The HD values calculated yielded comparable results with the smallest mean values for the left lung (20.8 ± 1.5), right lung (21.2 ± 10.5), left kidney (17.5 ± 12.9), and trachea (7.6 ± 6.9) and interquartile ranges between 4 mm (25th quartile for trachea) and 25 mm (75th quartile for right lung). The right kidney (22.9 ± 12.9), heart (31.2 ± 10.2), and esophagus (30.6 ± 11.4) showed interquartile ranges of approx. 15–25 mm, 26–35 mm, and 25–35 mm, respectively. The highest interquartile ranges were found for the liver and spinal cord ranging from approx. 24 to 50 mm and 6 to 47 mm, respectively. Of note, higher interquartile ranges and lower similarity values were not principally associated with data derived from postoperative patient datasets (in Fig. 3 corresponding results of postoperative data are marked as red dots).

### Qualitative assessment

Table 3 shows the expert scoring results and indicates that the reliability of the AS structures varied according to the OARs. The highest score was achieved in both lungs as OARs with 95% of all AS structures being considered as useful without correction (18 of 19 cases with score = 1). On the other hand, the esophagus and liver were considered not useful (score = 3, manual or semi-automatic contours were preferred) in 26 and 12% of the cases, respectively. For the trachea 90% of the AS objects were clinically useful without any correction, and one needed minor corrections (5%/5% with score = 2 or 3). In summary, the best results were obtained for air-containing tissue (lung, trachea). However, for the esophagus the quality of the automatically generated structures was poor.

**Table 3:** Results of qualitative assessment

Organ at risk	Expert scoring results in %			Mean score	Sample size
	Score = 1 (%)	Score = 2 (%)	Score = 3 (%)		
Left lung	94	0	6	1.12	17
Right lung	94	0	6	1.12	17
Trachea	88	6	6	1.17	18
Left kidney	79	21	0	1.21	14
Right kidney	61	31	8	1.46	13
Spinal cord	62	38	0	1.38	24
Heart	46	47	7	1.60	15
Liver	50	36	14	1.64	14
Esophagus	0	71	29	2.29	17

The case numbers in the qualitative assessment are higher than the ones in the quantitative assessment (see Discussion for further information)

**Fig. 4:**
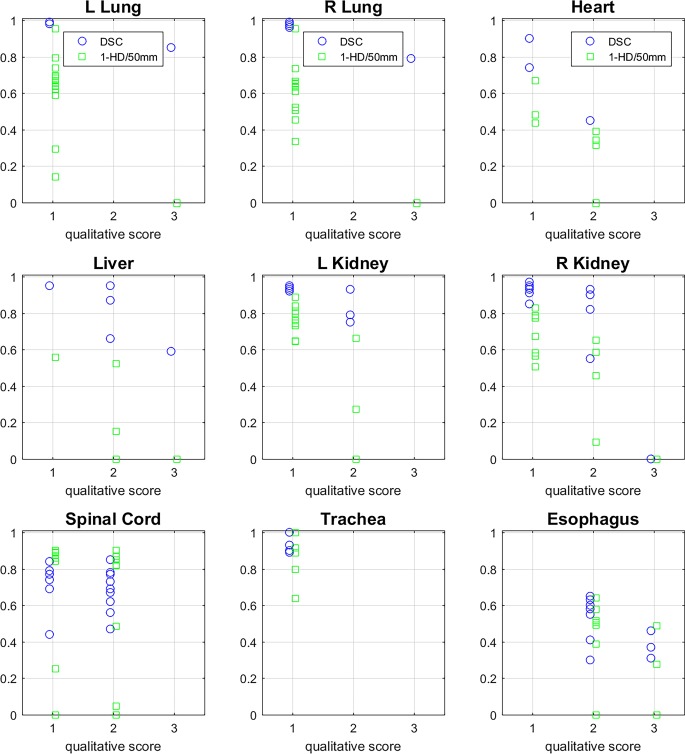
Quantitative vs. qualitative assessment, depiction of every DSC (*blue*) and HD (*green*) value for each organ sorted as a function of the related qualitative score (1, 2, 3). Since high DSC and low HD values are good, HD is displayed as 1‑(HD)/50 mm. *DSC* Dice similarity coefficient, *HD* Hausdorff distance, *L* left, *R* right

**Fig. 5:**
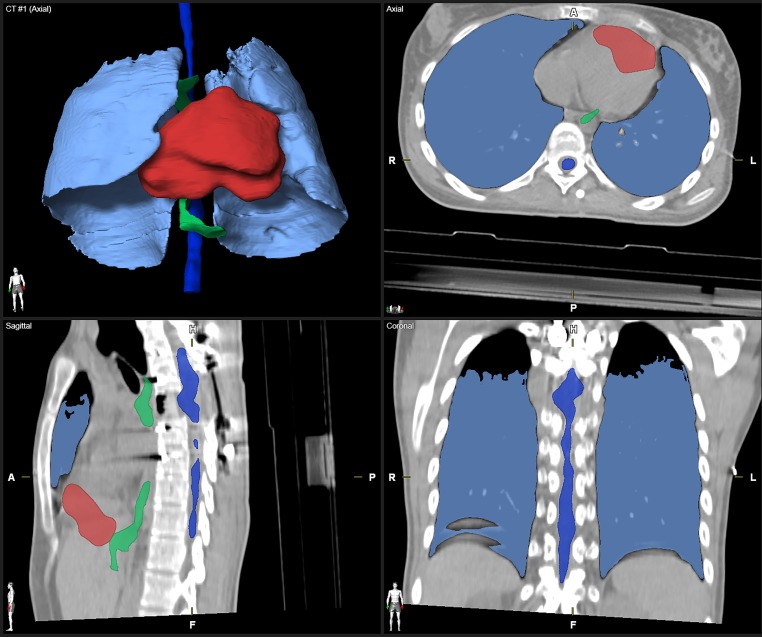
Example of a set of auto-segmented structures scored 3 in axial, coronal, sagittal view with three-dimensional reconstruction (postoperative CT). For illustrative purposes the visualization of auto-segmented objects was modified to show surfaces/volumes instead of contours.* DSC* Dice similarity coefficient, *HD* Hausdorff distance, *L* left, *R* right, *CT* Computed Tomography

**Fig. 6:**
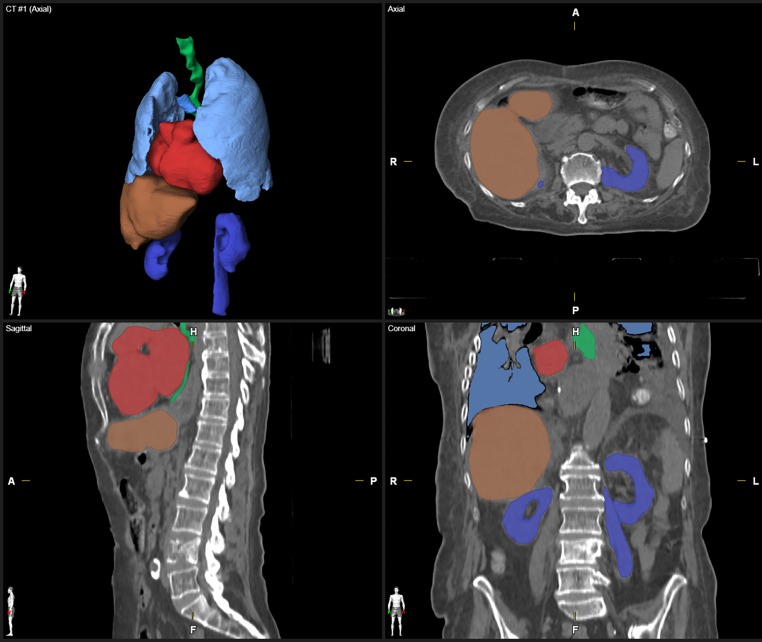
Another example of a not-accepted automatic segmentation (expert score = 3). In sagittal view direct contact of two organs (left kidney and left big psoas muscle) leads to a combined structure

In addition, the expert scoring results were graphically interrelated with the quantitative results of DSC and 1‑(HD)/50 mm values in order to support the quantitative assessment (Fig. 4). Principally, AS structures scored with 1 show higher DSCs and lower HDs than those scored with 2 or 3. However, in five cases with relatively high HDs for the spinal cord (>25 mm), clinical acceptance was still good (40% with score 1 and 60% with score 2, no score 3). In another patient, the left and right lung were scored with 3—the cause of these unacceptable results was a postoperative loss of four vertebrae (Fig. 5 left)—even though the DSCs showed acceptable results (0.85 and 0.79, respectively). The HDs in this case were >50 mm. One more example of a not-accepted AS of the left kidney is shown in Fig. 6. The auto-segmented contour of the left kidney totally involved the big psoas muscle because of direct contact of both organs (see sagittal view).

## Discussion

Auto-segmentation is a powerful tool for contouring in radiation oncology. Currently, several commercial software solutions are available (SPICE, ABAS, MIM, Velocity), which have been evaluated in the past [2, 7, 8]. It was concluded that the tools had similar performance (with regard to time-saving) with promising results (quality of AS contours). Difficulties were related to the interpretation of the quantitative parameters (DSC and HD). Jameson et al. demonstrated that these parameters are generally not able to provide a clear statement about the clinical usability of a certain contour [13]. Structures with small volumes may even show lower and more variable DSCs than structures with high volumes [17]. To support the quantitative evaluation, we have used the aforementioned expert scoring system as introduced by Zhu and coworkers [9] as an additional parameter in this investigation.

In our study, the AS of the OARs generated with the new Brainlab Anatomical Mapping software mostly showed good conformity with the RC (example: Figs. 1 and 2). Good results with high quality of the automatically generated structures were obtained in both lungs and trachea as OARs. These air-containing organs are characterized by high contrast to their surroundings. The expert scoring results determined in this study support these findings. Acceptance of the lungs and left kidney was quite good, having a score of 1 in >90 and >70% of cases, respectively (i.e., no edits needed). The heart, liver, and right kidney (we had two outliers with DSC <0.5) showed acceptable scores in this investigation, although scores were lower and the HD was higher than for the lungs and left kidney. The structures of these OARs were considered as acceptable without further editing required in more than half and up to three quarters of the cases. The number of structures that were scored as unacceptable was low for all of these OARs (<10%). Zhu et al. obtained comparable results in their investigation of the SPICE algorithm [9].

The poorest contouring quality was observed for the esophagus. However, manual contouring of the esophagus is difficult, even for experienced radiation oncologists. The long and highly variable mediastinal course of the esophagus, its variable diameter, the inconstant visibility of its lumen and the low contrast to its surroundings all together render the esophagus the most complex OAR in the thoracic region. As an outstanding example, Collier et al. even demonstrated a case with no overlap of the contour in single slices outlined by two different radiation oncologists ([4], Fig. 3). In our analysis, none of the auto-segmented esophagus contours were considered as useful without corrections, but almost 75% were acceptable with only minor corrections required (editing the Anatomical Mapping auto-segmentation was preferred over manual contouring).

The ambiguous results of the spinal cord (Fig. 4, good qualitative acceptance but high HD) correlate with the interobserver variabilities in contouring of the spinal cord due to its elongated shape. Nieder et al. demonstrated that even for SBRT is the interpretation of the spinal cord heterogeneous [18]. In particular, variation of the cranial and caudal borders with inclusion of parts of the brainstem or the cauda equina can cause high HD (maximum distance of surface points), while the effect on the volume is less crucial (DSC). Additionally, the evaluated software is able to distinguish between the spinal cord and spinal canal, but only the spinal cord was tested in our study with qualitative scoring results ranging between 1 and 2 (no spinal cord was scored 3). This also illustrates that DSC and HD alone are not able to measure the clinical acceptance of AS. Factors such as proximity to the planning target volume (PTV), the subscribed dose, and applied radiation method could influence the Radioation-Oncologist´s (RO) accuracy in OAR delineation and the acceptance of AS. In the qualitative review, we rated every AS structure as if it was to be used for SBRT, even though not all RCs were delineated for this purpose (Table 1).

One of the biggest shortcomings of this study is that this expert scoring was performed by only one RO and that the “minor edits” necessary to have a structure scored 2 upgraded to a scored 1 were not applied. A second big shortcoming is the small sample size. The retrospective design of our study offered surprisingly low numbers of contoured livers, hearts, and tracheae. Even though these OARs were included in more scans, they were not always outlined during clinical routine (no RCs available). By contrast, since AS was performed for all structures the qualitative assessment was done with the full number of cases (resulting in larger numbers in Table 3 compared with Table 2). The OARs of the whole pelvic region (prostate, bladder, rectum, hip joints, seminal vesicles, penile bulb) were only included in the images of one case (and consequently excluded from this study). Here, we recommend more careful a priori statistical considerations for designing a larger, prospective study. The scans of different body regions should be standardized and evaluated separately. As an example, for the whole pelvic region images of prostate patients would contain all the AS pelvic structures provided [8] and in the thoracic region scans of breast cancer patients would build a much more homogeneous cohort [19]. The inclusion criteria of our study (RT for spinal metastasis in the period from November 2016 through June 2017) proved to be too simple.

Owing to the retrospective nature of our investigation, we had to omit another interesting test: the analysis of segmentation time (manual contouring compared with auto-segmentation). However, we see a large potential of AS for optimizing the workflow. After PACS import, the tested software starts automatically with AS as a background routine (5–20 min). Afterwards every structure must be evaluated (and corrected when necessary) by the RO before dose-planning is possible. To prospectively evaluate the clinical benefit of this AS method compared with currently established manual or semi-automatic contouring approaches, further studies are necessary.

## Conclusions

The study has limitations regarding the data collection (low case numbers, inhomogeneous cohort). As a first pilot feasibility study, comparison of auto-segmentation and reference contours was done via quantitative and qualitative assessment. The evaluated organs at risk were the lungs, trachea, esophagus, heart, liver, kidneys, and spinal cord. Regarding these OARs, (excluding the esophagus) one can reasonably assume that AS software produces highly conformal structure sets (hypothesis a) and is ready for clinical usage (hypothesis b). So far, the evaluated software (Anatomical Mapping 1.0) is embedded in treatment planning for spinal metastasis, but implementation in clinical routine for the whole thoracic and abdominal region (and not only for spinal metastasis) is possible with individual workflow-optimizations. The estimated great potential of time-saving and standardization of OAR contours was not yet tested.
